# Supplementary material for: Using triangulation to evaluate findings from random-intercept cross-lagged panel models: An application with data on curiosity and creativity
Source: PLoS One. 2025 Dec 4;20(12):e0337928. doi: 10.1371/journal.pone.0337928 (PMC12677492; doi:10.1371/journal.pone.0337928)
Supplement: Attachment — Submitted filename: Triangulation_Response_R1.docx [file pone.0337928.s001.docx]

PONE-D-24-14717

Using triangulation to evaluate findings from random-intercept cross-lagged panel models: An application with data on curiosity and creativity

PLOS ONE

Dear Dr. Sorjonen,

Thank you for submitting your manuscript to PLOS ONE. After careful consideration, we feel that it has merit but does not fully meet PLOS ONE’s publication criteria as it currently stands. Therefore, we invite you to submit a revised version of the manuscript that addresses the points raised during the review process.

We look forward to receiving your revised manuscript.

Kind regards,

Vanessa Carels
Staff Editor
PLOS ONE

Reviewers' comments:

Reviewer's Responses to Questions

**Comments to the Author**

1. Is the manuscript technically sound, and do the data support the conclusions?

The manuscript must describe a technically sound piece of scientific research with data that supports the conclusions. Experiments must have been conducted rigorously, with appropriate controls, replication, and sample sizes. The conclusions must be drawn appropriately based on the data presented.

Reviewer #1: Partly

Reviewer #2: Yes

Reviewer #3: Yes

Response: We hope the amendments have increased the technical soundness of the manuscript.

2. Has the statistical analysis been performed appropriately and rigorously?

Reviewer #1: Yes

Reviewer #2: Yes

Reviewer #3: Yes

3. Have the authors made all data underlying the findings in their manuscript fully available?

The [PLOS Data policy](https://eur01.safelinks.protection.outlook.com/?url=http%3A%2F%2Ftrack.editorialmanager.com%2FCL0%2Fhttp%3A%252F%252Fwww.plosone.org%252Fstatic%252Fpolicies.action%2523sharing%2F1%2F010f01999f36850a-9db50b6d-26cc-466e-953e-f91b00f46f89-000000%2Fj6Gfwk1Pf5yaG23Nl_QeB3EYcFJ29xk2pzQfcg_qpcg%3D230&data=05%7C02%7Ckimmo.sorjonen%40ki.se%7Cc89463c390444a7b16bc08de00d13a33%7Cbff7eef1cf4b4f32be3da1dda043c05d%7C0%7C0%7C638949096099297820%7CUnknown%7CTWFpbGZsb3d8eyJFbXB0eU1hcGkiOnRydWUsIlYiOiIwLjAuMDAwMCIsIlAiOiJXaW4zMiIsIkFOIjoiTWFpbCIsIldUIjoyfQ%3D%3D%7C0%7C%7C%7C&sdata=h1SvBsblnjQ0RtXXrQcFj8KQQ765XaT5%2FCZN%2FC3E%2BtM%3D&reserved=0) requires authors to make all data underlying the findings described in their manuscript fully available without restriction, with rare exception (please refer to the Data Availability Statement in the manuscript PDF file). The data should be provided as part of the manuscript or its supporting information, or deposited to a public repository. For example, in addition to summary statistics, the data points behind means, medians and variance measures should be available. If there are restrictions on publicly sharing data—e.g. participant privacy or use of data from a third party—those must be specified.

Reviewer #1: No

Reviewer #2: Yes

Reviewer #3: Yes

Response: As we say in the manuscript, data used by Ma and Wei, and reanalyzed by us, is available at the Open Science Framework (<https://osf.io/d48xa/>) (line 135). Our analytic script, which downloads data used by Ma and Wei and then conducts all analyses reported in our manuscript, is available at the Open Science Framework (<https://osf.io/rk4ne/>) (line 205). Anyone can download our script, run it, and receive the same results as reported in our manuscript.

4. Is the manuscript presented in an intelligible fashion and written in standard English?

PLOS ONE does not copyedit accepted manuscripts, so the language in submitted articles must be clear, correct, and unambiguous. Any typographical or grammatical errors should be corrected at revision, so please note any specific errors here.

Reviewer #1: Yes

Reviewer #2: Yes

Reviewer #3: Yes

5. Review Comments to the Author

Please use the space provided to explain your answers to the questions above. You may also include additional comments for the author, including concerns about dual publication, research ethics, or publication ethics. (Please upload your review as an attachment if it exceeds 20,000 characters)

**Reviewer #1:**

1. General Assessment
The manuscript presents a relevant contribution to the literature by examining the applicability of the random-intercept cross-lagged panel model (RI-CLPM) in analysing the longitudinal relationship between curiosity and creativity. The study proposes critically examining this model, employing triangulation with different analytical approaches, such as latent change models and multilevel regression. The study's main conclusion challenges previous findings that suggest a bidirectional causal relationship between curiosity and creativity, arguing that such associations may be spurious due to common latent factors. Despite the topic's relevance, methodological and interpretation aspects of the results can be improved to strengthen the robustness of the conclusions.

Response: We hope our amendments have strengthened the robustness of the conclusions.

2. Theoretical Foundation and Justification
The literature review presents a solid coverage of cross-lagged models, particularly the RI-CLPM, and their limitations in terms of causal inference. However, it could be expanded to include a more in-depth discussion on the suitability of the triangulation model in psychological research, exploring previous examples of its application. In addition, a greater alignment between the theoretical review and the formulation of hypotheses is recommended, making the relationship between the triangulated approaches and the gaps the study aims to fill more explicit.

Response: We have added the following (lines 109-114):

We have previously used triangulation, i.e., analyzed data with alternative models, to challenge conclusions based on findings from analyses with the traditional CLPM [[e.g., 9–11]](https://www.zotero.org/google-docs/?hycJhF). Moreover, in a recent study we used triangulation to scrutinize and challenge conclusions of causal effects of academic self-concept (i.e., self-perceived academic competence) on academic achievement based on findings from analyses with the RI-CLPM [[12]](https://www.zotero.org/google-docs/?31x2Hu).

3. Methodology
The choice of triangulation with multiple statistical models is a strength of the manuscript, as it increases the reliability of the findings. However, there is a need for greater clarity in the justification for selecting auxiliary models, such as the latent change model and multilevel regression with mean-centered scores. In addition, the description of the data used, which came from a sample of employees from three companies in Hong Kong, could be expanded to consider possible cultural and sampling biases that may impact the generalizability of the findings.

Response: We have added the following (lines 193-202):

Analyzing models 2 and 5 were in line with proposals that time-reversed analyses may be used to identify potential statistical artifacts [[19,20]](https://www.zotero.org/google-docs/?UPBuWh). Previous analyses of data including an unquestionable causal effect (of adding stones in a container on the total weight of the container) found consistent effects in models 1-6 (i.e., effects in line with the predictions outlined above), suggesting that these models may be used to discriminate between true causal and spurious effects [[21]](https://www.zotero.org/google-docs/?WRzFTz). Model 7 (the MoSLA) was used in two recent studies and suggested that longitudinal data on academic self-concept and achievement [[12]](https://www.zotero.org/google-docs/?82WI3c) and on executive deficits and psychopathology [[22]](https://www.zotero.org/google-docs/?gfCQrb), respectively, may have been generated without any direct effects between these constructs and, consequently, that previous causal claims could be challenged.

As we say in the manuscript (lines 393-396):

all participants were employees at three firms in Hongkong, China, and it is unclear to what degree the present findings, that longitudinal associations between curiosity and creativity probably were spurious, generalizes to other cultural, social, and economical contexts.

We have now added the following (lines 396-402):

Hence, our findings may have been affected by cultural and sampling biases. However, we see no reason to believe that such biases would selectively have affected all models except the original RI-CLPM. Therefore, we do not believe that it would be tenable to argue that due to possible cultural and sampling biases, we should trust findings from the RI-CLPM, suggesting increasing prospective within-individual effects between curiosity and creativity, and dismiss contradictory findings from the other analyzed models.

4. Analysis of the Results
The results indicate that only the RI-CLPM suggests significant cross-lagged effects between curiosity and creativity, while the other models provide contrary or null evidence. The introduction of the model of spurious longitudinal associations (MoSLA) as an alternative explanatory tool is an interesting innovation, but could be better supported. Furthermore, the interpretation of the findings could include a more detailed discussion of the possible impacts of variability in individual scores and the sensitivity of the models to the characteristics of the data used.

Response: We have added the following (lines 198-202):

Model 7 (the MoSLA) was used in two recent studies and suggested that longitudinal data on academic self-concept and achievement [[12]](https://www.zotero.org/google-docs/?82WI3c) and on executive deficits and psychopathology [[22]](https://www.zotero.org/google-docs/?gfCQrb), respectively, may have been generated without any direct effects between these constructs and, consequently, that previous causal claims could be challenged.

Variability in individual scores is a prerequisite for associations and effects. Without variability, i.e., if all individuals would have the same score, it would not be possible to estimate associations or effects.

Moreover, as we say in the manuscript:

we know that a positive effect (indicating a decreasing effect) in models 2 and 5 is exactly what could be expected if curiosity and creativity were measured with less than perfect reliability (which should be very likely) and, therefore, susceptible to regression to the mean. Moreover, we also know that if measures of two constructs, e.g., curiosity and creativity, are affected by some common state factor (which also should be very likely), concurrent correlations (*r_X1,Y1_*) will tend to be stronger than cross-lagged correlations (*r_X1,Y2_*) which, according to Equation 1 [[27]](https://www.zotero.org/google-docs/?oX5G08), would result in a negative effect of X_1_ (e.g., initial curiosity) on the Y_2_-Y_1_ (e.g., subsequent - initial creativity) difference. (lines 348-356)

The used measures, self-rated for curiosity and supervisor-rated for creativity, may not have been optimal and susceptible to measurement errors, bias, etc. However, it is important to bear in mind that the same data were used across analyzed models in the present study. Hence, possible shortcoming in measurements were constant across the analyzed models and could, consequently, not explain why the models indicated simultaneous and paradoxical increasing, decreasing, and null effects between curiosity and creativity. (lines 403-408)

5. Discussion and Contribution
The discussion section correctly highlights the challenges in causal inference in longitudinal models and reinforces the importance of triangulated approaches. However, it would be beneficial to contextualize the findings within the psychological research field and apply these results in practice to future studies on creativity and curiosity. Furthermore, the suggested research agenda could include more specific recommendations on when and how triangulation should be used to analyze longitudinal data.

Response: We have added the following (lines 289-295):

The present divergent findings suggest that the data analyzed by Ma and Wei, and reanalyzed by us, offer no support to Ma and Wei’s hypothesis that curiosity fosters creativity because it improves individuals’ ability to develop original and useful ideas. Neither does the data appear to offer support to Ma and Wei’s hypothesis, based on the information-gap theory [[27]](https://www.zotero.org/google-docs/?ljhrIw), that creativity promotes curiosity because creativity prompts individuals to gather and scrutinize diverse information, make novel associations, gain fresh perspectives, and yearn for new knowledge.

Moreover, as we say in the manuscript (lines 372-374):

We believe that the present study, and the available analytic script (<https://osf.io/rk4ne/>), could be used as a model/tutorial by researchers wishing to scrutinize results from the RI-CLPM,

We have now added the following (lines 374-378):

i.e., when analyzing within-individual effects between two longitudinally measured constructs. The method of triangulation could be used with data on curiosity and creativity as well as observational (i.e., non-experimental) data on other constructs, both in psychology and other areas of research. Interested researchers are welcome to contact the corresponding author (KS) for advice and assistance.

6. Conclusion and Recommendations
The manuscript represents a valuable contribution to understanding the limitations of cross-lagged models and promoting triangulation in longitudinal analyses. However, the following improvements are recommended before acceptance for publication:

- Expand the theoretical review to contextualize the use of triangulation in psychology better;

Response: We have added the following (lines 109-114):

We have previously used triangulation, i.e., analyzed data with alternative models, to challenge conclusions based on findings from analyses with the traditional CLPM [[e.g., 9–11]](https://www.zotero.org/google-docs/?hycJhF). Moreover, in a recent study we used triangulation to scrutinize and challenge conclusions of causal effects of academic self-concept (i.e., self-perceived academic competence) on academic achievement based on findings from analyses with the RI-CLPM [[12]](https://www.zotero.org/google-docs/?31x2Hu).

- Justify in more detail the choice of auxiliary models used in the analysis;

Response: We have added the following (lines 193-202):

Analyzing models 2 and 5 were in line with proposals that time-reversed analyses may be used to identify potential statistical artifacts [[19,20]](https://www.zotero.org/google-docs/?UPBuWh). Previous analyses of data including an unquestionable causal effect (of adding stones in a container on the total weight of the container) found consistent effects in models 1-6 (i.e., effects in line with the predictions outlined above), suggesting that these models may be used to discriminate between true causal and spurious effects [[21]](https://www.zotero.org/google-docs/?WRzFTz). Model 7 (the MoSLA) was used in two recent studies and suggested that longitudinal data on academic self-concept and achievement [[12]](https://www.zotero.org/google-docs/?82WI3c) and on executive deficits and psychopathology [[22]](https://www.zotero.org/google-docs/?gfCQrb), respectively, may have been generated without any direct effects between these constructs and, consequently, that previous causal claims could be challenged.

- Deepen the discussion on the implications of the findings for research on creativity and curiosity;

Response: We have added the following (lines 289-295):

The present divergent findings suggest that the data analyzed by Ma and Wei, and reanalyzed by us, offer no support to Ma and Wei’s hypothesis that curiosity fosters creativity because it improves individuals’ ability to develop original and useful ideas. Neither does the data appear to offer support to Ma and Wei’s hypothesis, based on the information-gap theory [[27]](https://www.zotero.org/google-docs/?ljhrIw), that creativity promotes curiosity because creativity prompts individuals to gather and scrutinize diverse information, make novel associations, gain fresh perspectives, and yearn for new knowledge.

- Explore more clearly the limitations and potential biases of the sample used.

Response: As we say in the manuscript (lines 393-396):

all participants were employees at three firms in Hongkong, China, and it is unclear to what degree the present findings, that longitudinal associations between curiosity and creativity probably were spurious, generalizes to other cultural, social, and economical contexts.

We have now added the following (lines 396-402):

Hence, our findings may have been affected by cultural and sampling biases. However, we see no reason to believe that such biases would selectively have affected all models except the original RI-CLPM. Therefore, we do not believe that it would be tenable to argue that due to possible cultural and sampling biases, we should trust findings from the RI-CLPM, suggesting increasing prospective within-individual effects between curiosity and creativity, and dismiss contradictory findings from the other analyzed models.

**Reviewer #2:**

This study re-examines prior findings that curiosity and creativity mutually reinforce each other using the random-intercept cross-lagged panel model (RI-CLPM). Acknowledging potential bias in RI-CLPM results, the authors apply triangulation with alternative methods, including a latent change score model (LCSM), multilevel regression with person-mean centering, and the novel Model of Spurious Longitudinal Associations (MoSLA). Contrary to the RI-CLPM, other models did not support mutual reinforcement, and MoSLA suggested the observed links may be spurious, possibly driven by shared traits or common state factors. The work underscores the need for model comparison in longitudinal research and offers analytic scripts as a resource for methodological scrutiny.

Response: We agree with this summary of the manuscript.

**Reviewer #3:**

In this paper, the authors reanalyzed data previously analyzed and used to draw a strong conclusion about the relationship between creativity and curiosity over time. Instead of using one model, these authors analyzed three models using both covariance (i.e., structural equation modeling) and mean-based (i.e., multilevel regression) methods as well as a fourth (i.e., seventh) model. Two of the models are similar expressions as the original data analysis. The version that matches the original analysis faithfully recreates the results, but no other models find the same results as the original analysis, and some find results that contradict the original analysis. The authors conclude that, on the weight of the analytical evidence, a strong association between creativity and curiosity across time cannot be supported.

This paper is clever and admirably succinct. The modeling choices are excellent, and the methods used are sufficient. I would love to see them take the leap into the Bayesian world, where comparing and integrating across models comes naturally, but that should not diminish this paper at all. The authors could have made different design decisions through the paper (and, indeed, I might have), but their design decisions are reasonable and lead to an excellent paper. I believe that this paper can be published as is. In case the authors would like things to work on, here are a few:

1) The weight of water example is good, but it can be very dense and non-intuitive. I imagine a lot of time was spent coming up with this example, and I do think it is good. Maybe a figure for this instead of the triangulation figure (which I think is much more intuitive for people) would be good. If not, maybe consider dividing that one big paragraph into smaller paragraphs and helping the reader move through it a little better. Insertion of connective tissue and more aids to the reader could help here.

Response: We have divided the paragraph into smaller paragraphs. We have also added a new Figure 2 as well as the following:

For example, with the data in Fig 2A, the effect of amount of poured water (milliliters) on weight at T2 (grams) when adjusting for weight at T1 (i.e., conditioning on the same weight at T1) would equal *b* = 1. (lines 81-84)

With the data in Fig 2B, the effect of amount of poured water (milliliters) on weight at T1 (grams) when adjusting for weight at T2 (i.e., conditioning on the same weight at T2) would equal *b* = -1. (lines 90-92)

With the data in Fig 2A and 2B, the effect of amount of poured water (milliliters) on the weight at T2 - weight at T1 difference (grams) would equal *b* = 1. (lines 95-97)

2) Similarly, the first paragraph of the Results section is quite dense. Much of this is because of the repetition of "within-individual residual". As an applied statistician, I value the precision. As a reader, it makes it hard to focus on the things that actually matter. This might benefit from breaking it out into multiple paragraphs and really guiding the reader through it. Everything should be obvious. Maybe, since you've now spent so long getting them to understand the water example possibly even with a figure, you can tie everything very explicitly to the water example. In this water case, what would this finding imply? That might help people really see what fails.

Response. We have divided the paragraph into smaller paragraphs. We have also added the following:

This corresponds to the amount of poured water having, as expected, a positive effect on the weight of cups at T2 when conditioning on the same weight at T1 (Fig 2A). (lines 213-215)

This positive effect would correspond to the amount of poured water having, contrary to expectations illustrated in Fig 2B, a positive effect on the weight of cups at T1 when conditioning on the same weight at T2. (lines 229-231)

These negative effects would correspond to the amount of poured water having, contrary to expectations, a negative effect on the weight at T2 - weight at T1 difference of cups (Fig 2). (lines 235-237)

3) It would be possible to reconceive of this paper as an explicit tutorial. I am not suggesting the authors do that but thinking of it that way may help them see where they might expand on a few points.

A minor point (but especially important if they want this to function as a tutorial): I believe that std.nox has been deprecated from the parameterEstimates function in lavaan. I was able to recreate the analysis anyway, but others may not be able to. You should make sure this works no matter what. Maybe use renv to create a virtual environment that ensures everyone gets the same versions of the packages and that it will work anywhere.

Response: Thanks for the tip. We have changed “std.nox” to “std.all” which is compatible with the latest (0.6-20) version of lavaan. We have also uploaded a zipped project file with renv activated and a Read_Me-file with instructions (<https://osf.io/rk4ne/>).

Note that I do not comment on writing directly, provide line edits, or copyedit in my reviews.

Curtis Atkisson
