# Supplementary material for: Using triangulation to evaluate findings from random-intercept cross-lagged panel models: An application with data on curiosity and creativity
Source: PLoS One. 2025 Dec 4;20(12):e0337928. doi: 10.1371/journal.pone.0337928 (PMC12677492; doi:10.1371/journal.pone.0337928)
Supplement: Attachment — Submitted filename: Triangulation_Response_R2.docx [file pone.0337928.s002.docx]

PONE-D-24-14717R1

Using triangulation to evaluate findings from random-intercept cross-lagged panel models: An application with data on curiosity and creativity

PLOS ONE

Dear Dr. Sorjonen,

Thank you for submitting your manuscript to PLOS ONE. After careful consideration, we feel that it has merit but does not fully meet PLOS ONE’s publication criteria as it currently stands. Therefore, we invite you to submit a revised version of the manuscript that addresses the points raised during the review process.

The paper is poorly written; many points and claims are incomprehensible.

1. The triangulation problem and circle illustration in Fig. 1 make little sense and are disconnected from the statistical model used by the authors. For example, if the points are in a three-dimensional space, the author’s conclusion is wrong.  The authors do not solve a location problem, and therefore, this illustration does not work. I guess the authors meant that more information implies a more accurate answer or solution— a trivial statement.

Response: Although the point that more information allows more accurate answers may seem trivial, it appears not to be appreciated by many researchers. For example, it is quite common to conclude, as in the study by Ma and Wei (2023) that we challenge, causal effects based on cross-lagged effects without requiring any additional information. The point we are making with the triangulation example is that just as we should not infer a location based on its distance from another (known) location (something we assume/hope that all readers would agree with), we should not infer causality from a cross-lagged effect in observational data. We hope that this may open some eyes among researchers who have been lured into believing that cross-lagged panel models (with or without random intercepts) may, magically, reveal causality in observational data. We have added the following (line 41);

(in a two-dimensional space)

2. The statistical model, the heart of the discussion, is not defined. It is impossible to judge the validity of the conclusions and results until the model, along with all the involved variables depicted in Fig. 3, is mathematically defined in equation form (if the authors feel the model is too complicated, it may be presented in the Supplementary Materials file). The reader does not know what exactly the model is. The table with all estimated coefficients, along with standard errors and p-values, must be presented as well.

Response: We believe it to be standard practice to present structural equation models (SEM) as path diagrams rather than as equations. See, for example, Sorjonen et al. (2025) for a recent example by us and Toyoshima et al. (2020) for an example published in PLOS ONE. Such path-diagrams can also be found in very prestigious journals (e.g., Gosling et al., 2022). However, we have included documents with all effects in equation form as well as all output from the analyzed models (including standard errors and p-values) in our supplementary. We have added the following:

(regression equations for effects included in these models can be found in the document “Supplementary_Equations” available at <https://osf.io/rk4ne/>) (lines 148-150).

(full output for all models, including standard errors, p-values, etc., can be found in the document “Supplementary_Output” available at <https://osf.io/rk4ne/>) (lines 221-222).

As a general critique, the paper lacks transparency regarding its modeling assumptions, making it difficult to assess the validity of the results.

Response: We have made the analytic script publicly available (see line 216). Anyone with access to a computer and the internet can download the script, run it (with the statistical freeware R), and receive the same results as we did. This was confirmed in the previous round by reviewer # 3. Now we have also included documents with all effects in equation form as well as all output from the analyzed models (including standard errors and p-values) in our supplementary. We have added the following:

(regression equations for effects included in these models can be found in the document “Supplementary_Equations” available at <https://osf.io/rk4ne/>) (lines 148-150).

(full output for all models, including standard errors, p-values, etc., can be found in the document “Supplementary_Output” available at <https://osf.io/rk4ne/>) (lines 221-222).

Please submit your revised manuscript by Dec 12 2025 11:59PM. If you will need more time than this to complete your revisions, please reply to this message or contact the journal office at [plosone@plos.org](mailto:plosone@plos.org). When you're ready to submit your revision, log on to <https://www.editorialmanager.com/pone/> and select the 'Submissions Needing Revision' folder to locate your manuscript file.

Please include the following items when submitting your revised manuscript:

- A rebuttal letter that responds to each point raised by the academic editor and reviewer(s). You should upload this letter as a separate file labeled 'Response to Reviewers'.
- A marked-up copy of your manuscript that highlights changes made to the original version. You should upload this as a separate file labeled 'Revised Manuscript with Track Changes'.
- An unmarked version of your revised paper without tracked changes. You should upload this as a separate file labeled 'Manuscript'.

If you would like to make changes to your financial disclosure, please include your updated statement in your cover letter. Guidelines for resubmitting your figure files are available below the reviewer comments at the end of this letter.

If applicable, we recommend that you deposit your laboratory protocols in protocols.io to enhance the reproducibility of your results. Protocols.io assigns your protocol its own identifier (DOI) so that it can be cited independently in the future. For instructions see: [https://journals.plos.org/plosone/s/submission-guidelines#loc-laboratory-protocols](https://eur01.safelinks.protection.outlook.com/?url=https%3A%2F%2Ftrack.editorialmanager.com%2FCL0%2Fhttps%3A%252F%252Fjournals.plos.org%252Fplosone%252Fs%252Fsubmission-guidelines%2523loc-laboratory-protocols%2F1%2F010f019a2dfee138-426a8e02-a16f-435d-859d-d48cb78bac9e-000000%2FBEYCa3ByAsmuogiXF3DiERV0SZK5KPSzJXX33LhiT0U%3D235&data=05%7C02%7Ckimmo.sorjonen%40ki.se%7C68b2842dc3814e61266d08de169aacf0%7Cbff7eef1cf4b4f32be3da1dda043c05d%7C0%7C0%7C638973050756540797%7CUnknown%7CTWFpbGZsb3d8eyJFbXB0eU1hcGkiOnRydWUsIlYiOiIwLjAuMDAwMCIsIlAiOiJXaW4zMiIsIkFOIjoiTWFpbCIsIldUIjoyfQ%3D%3D%7C0%7C%7C%7C&sdata=unDlzpg7qmd4QIm%2Fug56ANIrSs%2Fz3x3DjiPE5MdAA3s%3D&reserved=0). Additionally, PLOS ONE offers an option for publishing peer-reviewed Lab Protocol articles, which describe protocols hosted on protocols.io. Read more information on sharing protocols at [https://plos.org/protocols?utm_medium=editorial-email&utm_source=authorletters&utm_campaign=protocols](https://eur01.safelinks.protection.outlook.com/?url=https%3A%2F%2Ftrack.editorialmanager.com%2FCL0%2Fhttps%3A%252F%252Fplos.org%252Fprotocols%253Futm_medium%3Deditorial-email%2526utm_source%3Dauthorletters%2526utm_campaign%3Dprotocols%2F1%2F010f019a2dfee138-426a8e02-a16f-435d-859d-d48cb78bac9e-000000%2Fb7q2HhVUSiEs2RbzOQyxkQ54_EtroD3SysSibUdDzlo%3D235&data=05%7C02%7Ckimmo.sorjonen%40ki.se%7C68b2842dc3814e61266d08de169aacf0%7Cbff7eef1cf4b4f32be3da1dda043c05d%7C0%7C0%7C638973050756575289%7CUnknown%7CTWFpbGZsb3d8eyJFbXB0eU1hcGkiOnRydWUsIlYiOiIwLjAuMDAwMCIsIlAiOiJXaW4zMiIsIkFOIjoiTWFpbCIsIldUIjoyfQ%3D%3D%7C0%7C%7C%7C&sdata=U9%2FUFJzC1TDhR4iP2%2B%2B5DW8j2TyMmrfTbJB5gGYtG6k%3D&reserved=0).

We look forward to receiving your revised manuscript.

Kind regards,

Eugene Demidenko, Ph.D.
Academic Editor
PLOS ONE

Journal Requirements:

If the reviewer comments include a recommendation to cite specific previously published works, please review and evaluate these publications to determine whether they are relevant and should be cited. There is no requirement to cite these works unless the editor has indicated otherwise. 

Additional Editor Comments:

The paper is poorly written; many points and claims are incomprehensible.

1. The triangulation problem and circle illustration in Fig. 1 make little sense and are disconnected from the statistical model used by the authors. For example, if the points are in a three-dimensional space, the author’s conclusion is wrong. The authors do not solve a location problem, and therefore, this illustration does not work. I guess the authors meant that more information implies a more accurate answer or solution— a trivial statement.

Response: Although the point that more information allows more accurate answers may seem trivial, it appears not to be appreciated by many researchers. For example, it is quite common to conclude, as in the study by Ma and Wei (2023) that we challenge, causal effects based on cross-lagged effects without requiring any additional information. The point we are making with the triangulation example is that just as we should not infer a location based on its distance from another (known) location (something we assume/hope that all readers would agree with), we should not infer causality from a cross-lagged effect in observational data. We hope that this may open some eyes among researchers who have been lured into believing that cross-lagged panel models (with or without random intercepts) may, magically, reveal causality in observational data. We have added the following (line 41);

(in a two-dimensional space)

2. The statistical model, the heart of the discussion, is not defined. It is impossible to judge the validity of the conclusions and results until the model, along with all the involved variables depicted in Fig. 3, is mathematically defined in equation form (if the authors feel the model is too complicated, it may be presented in the Supplementary Materials file). The reader does not know what exactly the model is. The table with all estimated coefficients, along with standard errors and p-values, must be presented as well.

Response: We believe it to be standard practice to present structural equation models (SEM) as path diagrams rather than as equations. See, for example, Sorjonen et al. (2025) for a recent example by us and Toyoshima et al. (2020) for an example published in PLOS ONE. Such path-diagrams can also be found in very prestigious journals (e.g., Gosling et al., 2022). However, we have included documents with all effects in equation form as well as all output from the analyzed models (including standard errors and p-values) in our supplementary. We have added the following:

(regression equations for effects included in these models can be found in the document “Supplementary_Equations” available at <https://osf.io/rk4ne/>) (lines 148-150).

(full output for all models, including standard errors, p-values, etc., can be found in the document “Supplementary_Output” available at <https://osf.io/rk4ne/>) (lines 221-222).

As a general critique, the paper lacks transparency regarding its modeling assumptions, making it difficult to assess the validity of the results.

Response: We have made the analytic script publicly available (see line 216). Anyone with access to a computer and the internet can download the script, run it (with the statistical freeware R), and receive the same results as we did. This was confirmed in the previous round by reviewer # 3. Now we have also included documents with all effects in equation form as well as all output from the analyzed models (including standard errors and p-values) in our supplementary. We have added the following:

(regression equations for effects included in these models can be found in the document “Supplementary_Equations” available at <https://osf.io/rk4ne/>) (lines 148-150).

(full output for all models, including standard errors, p-values, etc., can be found in the document “Supplementary_Output” available at <https://osf.io/rk4ne/>) (lines 221-222).

**References**

Gosling, W. D., Miller, C. S., Shanahan, T. M., Holden, P. B., Overpeck, J. T., & Van Langevelde, F. (2022). A stronger role for long-term moisture change than for CO_2_ in determining tropical woody vegetation change. *Science*, *376*(6593), 653–656. https://doi.org/10.1126/science.abg4618

Ma, J. (Yonas), & Wei, W. (2023). Curiosity causes creativity? Revealing the reinforcement circle between state curiosity and creativity. *The Journal of Creative Behavior*, jocb.606. https://doi.org/10.1002/jocb.606

Sorjonen, K., Melin, B., & Nilsonne, G. (2025). Inconclusive evidence for a prospective effect of academic self-concept on achievement: A simulated reanalysis and comment on Marsh et al. (2024). *Educational Psychology Review*, *37*(2), 30. https://doi.org/10.1007/s10648-025-10008-4

Toyoshima, K., Inoue, T., Masuya, J., Fujimura, Y., Higashi, S., Tanabe, H., & Kusumi, I. (2020). Structural equation modeling approach to explore the influence of childhood maltreatment in adults. *PLOS ONE*, *15*(10), e0239820. https://doi.org/10.1371/journal.pone.0239820

[Note: HTML markup is below. Please do not edit.]

Reviewers' comments:

Reviewer's Responses to Questions

**Comments to the Author**

1. If the authors have adequately addressed your comments raised in a previous round of review and you feel that this manuscript is now acceptable for publication, you may indicate that here to bypass the “Comments to the Author” section, enter your conflict of interest statement in the “Confidential to Editor” section, and submit your "Accept" recommendation.

Reviewer #3: All comments have been addressed

2. Is the manuscript technically sound, and do the data support the conclusions?

The manuscript must describe a technically sound piece of scientific research with data that supports the conclusions. Experiments must have been conducted rigorously, with appropriate controls, replication, and sample sizes. The conclusions must be drawn appropriately based on the data presented.

Reviewer #3: Yes

3. Has the statistical analysis been performed appropriately and rigorously?

Reviewer #3: Yes

4. Have the authors made all data underlying the findings in their manuscript fully available?

The [PLOS Data policy](https://eur01.safelinks.protection.outlook.com/?url=http%3A%2F%2Ftrack.editorialmanager.com%2FCL0%2Fhttp%3A%252F%252Fwww.plosone.org%252Fstatic%252Fpolicies.action%2523sharing%2F1%2F010f019a2dfee138-426a8e02-a16f-435d-859d-d48cb78bac9e-000000%2FlCJWa4G8w8WpLQtnorRMy0YY_fesMSNTfYx4CAKRLBE%3D235&data=05%7C02%7Ckimmo.sorjonen%40ki.se%7C68b2842dc3814e61266d08de169aacf0%7Cbff7eef1cf4b4f32be3da1dda043c05d%7C0%7C0%7C638973050756593274%7CUnknown%7CTWFpbGZsb3d8eyJFbXB0eU1hcGkiOnRydWUsIlYiOiIwLjAuMDAwMCIsIlAiOiJXaW4zMiIsIkFOIjoiTWFpbCIsIldUIjoyfQ%3D%3D%7C0%7C%7C%7C&sdata=XAUE%2Fr64y8Dt0fGVdm2z%2Blo%2FRn%2Bif%2B3JoYkIIG068Mg%3D&reserved=0) requires authors to make all data underlying the findings described in their manuscript fully available without restriction, with rare exception (please refer to the Data Availability Statement in the manuscript PDF file). The data should be provided as part of the manuscript or its supporting information, or deposited to a public repository. For example, in addition to summary statistics, the data points behind means, medians and variance measures should be available. If there are restrictions on publicly sharing data—e.g. participant privacy or use of data from a third party—those must be specified.

Reviewer #3: Yes

5. Is the manuscript presented in an intelligible fashion and written in standard English?

PLOS ONE does not copyedit accepted manuscripts, so the language in submitted articles must be clear, correct, and unambiguous. Any typographical or grammatical errors should be corrected at revision, so please note any specific errors here.

Reviewer #3: Yes

6. Review Comments to the Author

Please use the space provided to explain your answers to the questions above. You may also include additional comments for the author, including concerns about dual publication, research ethics, or publication ethics. (Please upload your review as an attachment if it exceeds 20,000 characters)

Reviewer #3: This revision satisfactorily addresses all my comments. This paper represents a good reanalysis of existing data.

7. PLOS authors have the option to publish the peer review history of their article ([what does this mean?](https://eur01.safelinks.protection.outlook.com/?url=https%3A%2F%2Ftrack.editorialmanager.com%2FCL0%2Fhttps%3A%252F%252Fjournals.plos.org%252Fplosone%252Fs%252Feditorial-and-peer-review-process%2523loc-peer-review-history%2F1%2F010f019a2dfee138-426a8e02-a16f-435d-859d-d48cb78bac9e-000000%2FG8kmrKC_JqFadRq07mxnwRfWvOedGfuZnz9SYWpui1U%3D235&data=05%7C02%7Ckimmo.sorjonen%40ki.se%7C68b2842dc3814e61266d08de169aacf0%7Cbff7eef1cf4b4f32be3da1dda043c05d%7C0%7C0%7C638973050756610290%7CUnknown%7CTWFpbGZsb3d8eyJFbXB0eU1hcGkiOnRydWUsIlYiOiIwLjAuMDAwMCIsIlAiOiJXaW4zMiIsIkFOIjoiTWFpbCIsIldUIjoyfQ%3D%3D%7C0%7C%7C%7C&sdata=fuQ3RWosBe61I62GqNjo66n4czcXExDhwh%2BZ3ZCPlps%3D&reserved=0)). If published, this will include your full peer review and any attached files.

If you choose “no”, your identity will remain anonymous but your review may still be made public.

**Do you want your identity to be public for this peer review?** For information about this choice, including consent withdrawal, please see our [Privacy Policy](https://eur01.safelinks.protection.outlook.com/?url=https%3A%2F%2Ftrack.editorialmanager.com%2FCL0%2Fhttps%3A%252F%252Fwww.plos.org%252Fprivacy-policy%2F1%2F010f019a2dfee138-426a8e02-a16f-435d-859d-d48cb78bac9e-000000%2Fx4704GM_AaszxnkkK8wU-vXSmbIvPhAp6h-MkIv8eLU%3D235&data=05%7C02%7Ckimmo.sorjonen%40ki.se%7C68b2842dc3814e61266d08de169aacf0%7Cbff7eef1cf4b4f32be3da1dda043c05d%7C0%7C0%7C638973050756625741%7CUnknown%7CTWFpbGZsb3d8eyJFbXB0eU1hcGkiOnRydWUsIlYiOiIwLjAuMDAwMCIsIlAiOiJXaW4zMiIsIkFOIjoiTWFpbCIsIldUIjoyfQ%3D%3D%7C0%7C%7C%7C&sdata=WU46mHOB2VmXUJtAd51dMTXAgdre0HAJbgoevwPZzqc%3D&reserved=0).

Reviewer #3: **Yes:** Curtis Atkisson
